# The Laundry Expenses of Hospitals: King Edward's Hospital Fund Statistics


**Published:** 1915-07-17

**Authors:** 


### THE LAUNDRY EXPENSES OF HOSPITALS.

### King Edward's Hospital Fund Statistics.*

A LARGE section of the annual return of the King's Fund is devoted to the statistics of the laundry expenses of hospitals. The expenditure under this heading of one hundred and six institutions is analysed and divided into three classes:—

A. Washing done on hospital premises.

B. Washing done off hospital premises.

C. Washing done partly on hospital premises and partly off.

There are eighteen hospitals in Class A; eighty-two in Class B, and six in Class C. It is stated that in order to enable the cost of washing at hospitals with laundries to be compared with the cost at hospitals which send their washing out, the whole cost of the laundry is taken into account. The figures given for cost per bed at hospitals with laundries include, therefore, various items (such as repairs, insurance, depreciation, interest on capital, and rent, rates, and taxes) which are omitted from the tables of cost per bed, etc., in the earlier sections of the report.

### A VITAL EXPLANATION.

This is important for two reasons; one is that when we examine the figures of hospitals working their own laundries, we are aware, in making comparisons, that the figures have been prepared on business lines and that all appropriate expenditure has been included; the other is that a clear warning is given that in the tables of the report showing the general expenditure of these institutions there is yet another unknown factor to be taken into consideration—namely, that there are expenses properly belonging to washing which are not included under the heading "Domestic." The matter is so important that it is worth while to quote the official footnote in full:—

"In order to facilitate comparisons between the domestic expenditure of hospitals where the washing is done on hospital premises and those where it is put out, the cost of the laundry in the former cases under the headings of 'Provisions,' 'Salaries,' 'Establishment Charges,' and 'Rent, Rates, and Taxes,' as shown in the washing returns published by these hospitals, has been transferred to the heading 'Domestic.' As the tables in Sections I. and II. of the report deal only with the figures of the income and expenditure accounts, the charges in respect of depreciation and interest on capital which form part of the cost of washing as shown in the washing returns are not included in the domestic expenditure. In the cases where the washing as shown in the washing returns is put out the domestic expenditure includes, of course, the whole of the charges of the laundry companies."

In previous papers we have pointed out that there is no general hospital auditor or supervising accountant. The division of expenditure between in-patients and out-patients and under the several headings of the expenditure account, is left to the discretion of the individual institutions, guided by certain general rules laid down by the Central Funds. That methods of allocation differ is not merely a matter of surmise we have shown in previous papers; that it is no less so in respect of the figures we are now examining, we hope to demonstrate; it is, therefore, too much to hope that opinions as to what sums shall be written off as depreciation and interest on capital are any less decided by individual judgment.

That said, we may proceed with criticism of the laundry section. In the eighteen hospitals with laundries, the cost per occupied bed under washing ranged from £3 18s. 11d. (Beckenham Cottage Hospital) to £18 14s. 7d. (London Fever Hospital). The lowest hospital of large size is the Seamen's Hospital at Greenwich, where the cost was £4 12s. 1d., and as the special character of the work at the Fever Hospital must exclude that institution from helpful comparison, it should be said that the next below the highest would be Poplar Hospital, with a cost of £10 7s. 11d. In passing it may be said that the laundry at the last-named institution was established within the last few years. The eighteen hospital laundries may be divided in respect of cost per bed as follows:—

One under £4; two under £5; three under £6; three under £7; three under £8; three under £9; one under £10; two over £10.

Of the 82 hospitals sending the washing out the lowest in cost is St. John's Hospital for Skin Diseases (£1 11s. 4d.), and the highest Queen Charlotte's Lying-in Hospital (£10 1s. 2d.). Two institutions were closed during 1913, the remainder present results as follows—One under £2, two under £3, nine under £4, twelve under £5, sixteen under £6, seventeen under £7, eleven under £8, nine under £9, two under £10, and one over £10. In the six hospitals sending some washing out and doing some at home the cost ranges from £4 4s. 2d. (London Lock) to £8 19s. 4d. (Home for Mothers, Lying-in). It is seen that in most cases over the three groups the cost per occupied bed under this heading is between £6 and £9.

COST OF WASHING AT FIVE LONDON GENERAL HOSPITALS WITH LAUNDRIES.

| Hospital | Occupied Beds | Approximate Percentage of Out-Patient Expenditure of the Whole Expenditure. | Average Weekly Number of Pieces | Cost per Occupied Bed £ s. d. |
|---|---|---|---|---|
| Guy's ......... | 527 | 8 per cent. | 24,871 | 7 13 8 |
| London ...... | 795 | 14 ,, | 53,735 | 6 19 11 |
| Middlesex ... | 337 | 3⅓ ,, | 16,006 | 8 5 10 |
| Royal Free ... | 143 | 12 ,, | 6,308 | 7 1 11 |
| Seamen's (Greenwich) | 222 | 8 ,, | 8,717 | 4 12 1 |

In the above tables, as in the others of the report, the cost of in-patients is separated from that of out-

* Previous articles have appeared on Oct. 10 and Dec. 2, 1914; and Jan. 23, Feb. 6 and 20, March 20, April 3, and May 15.

patients. In respect of the hospitals with laundries the comparative figures are given.

Of the methods of accounting employed by the hospitals quoted in the preceding table, it may be said that it is remarkable that the result shows such a large variation in the percentages allocated to the in- and out-patient departments. These are all general hospitals; the ratio between the number of out-patient attendances and the daily occupation of beds does not vary in any important respect. Yet we see that the Middlesex makes the division of the total cost $3\frac{1}{3}$ per cent. and $96\frac{2}{3}$ per cent., while the London employs the figures 14 per cent. and 86 per cent. The system of accounting in these instances must surely differ.

There are two instances of where the same laundry presumably serves two institutions. The Seamen's, Greenwich (cost £4 12s. 1d.) and the Seamen's, Albert Dock (cost £5 11s. 2d.), and the Middlesex (cost £8 5s. 10d.) and the Middlesex Cancer (cost £5 12s. 5d.). It would be interesting to know if the system of division in these cases has been carried out on the same lines.

### Cost and Number of Articles.

Another remarkable feature disclosed by the tables is the difference in the cost of laundry and in the number of articles used in hospitals of similar character and size. To illustrate this point the following comparisons are given, which are all taken from the "washing done off hospital premises" list:—

Comparative Table Showing Cost and Quantity of Laundry at Hospitals of Similar Character and Size.

| — | Occupied Beds | Out-Patient Attendances | Pieces Weekly | Total Cost | Cost per Bed | | |
|---|---|---|---|---|---|---|---|
| Larger General: | | | | £ | £ | s. | d. |
| St. Mary's ............ | 264 | 122,314 | 9,748 | 1,452 | 5 | 4 | 11 |
| University ............ | 281 | 105,217 | 7,717 | 1,202 | 4 | 0 | 3 |
| Smaller General: | | | | | | | |
| French .................. | 54 | 15,334 | 1,619 | 359 | 6 | 8 | 8 |
| Miller .................. | 55 | 91,625 | 2,196 | 453 | 7 | 6 | 11 |
| Women: | | | | | | | |
| Chelsea ............... | 48 | 8,810 | 2,740 | 361 | 7 | 10 | 11 |
| Samaritan Free ...... | 54 | 13,900 | 1,565 | 358 | 6 | 9 | 8 |
| Children: | | | | | | | |
| Belgrave ............... | 36 | 39,929 | 2,448 | 321 | 7 | 16 | 9 |
| Paddington Green... | 36 | 49,944 | 1,300 | 234 | 5 | 18 | 1 |
| Eye: | | | | | | | |
| Royal Eye ........... | 35 | 65,194 | 1,172 | 199 | 4 | 18 | 0 |
| Royal Westminster | 32 | 38,625 | 542 | 122 | 3 | 3 | 11 |
| Nervous Diseases: | | | | | | | |
| Hospital for Epilepsy ........... | 55 | 16,785 | 844 | 216 | 3 | 17 | 5 |
| West End............... | 50 | 29,046 | 1,765 | 284 | 5 | 6 | 5 |
| Lying-In: | | | | | | | |
| City of Lond[illegible] ...... | 42 | 10,802 | 2,360 | 288 | 6 | 17 | 2 |
| Queen Charlotte's... | 55 | 18,540 | 4,989 | 553 | 10 | 1 | 2 |

It is presumed that the weekly totals of articles used include all kinds of washing, not merely the flat things, such as draw-sheets, pillow slips, theatre towels, and the like, but also more elaborate pieces—screen covers, etc. This being so, the average cost per dozen articles is surprisingly low in some instances. This remark especially applies to three of the lying-in hospitals, which each return a rate of between 6d. and 7d. per dozen. Probably there is some special nursing reason for this result, as there would also be at the children's hospitals—each of them between 7d. and 9d. On the other hand, we find high rates elsewhere, and in this respect may be mentioned the Cancer (19.39d.), three ophthalmic institutions, and the Samaritan Free (each over 1s.).

### Perplexing Problems.

In turning to the preceding table we find some perplexing problems. Why should University College with 281 beds require only 7,717 articles weekly, while 9,748 are found necessary at St. Mary's, with seventeen fewer occupied beds. Chelsea finds a laundry to wash 2,740 pieces weekly at the same cost as the Samaritan Free, which uses 1,565 in the same period. Paddington Green, with the same number of beds, but receiving 10,000 more out-patient attendances than the Belgrave, uses only 1,300 pieces weekly at an annual cost of £234, against the 2,448 articles and £321 annual expenditure of the sister hospital.

However, it is probable that it can be successfully argued that none of the laundry figures in the statistical report are a reliable guide upon which to base judgment as to a hospital's economy or want of economy. One of the most important factors is not the annual cost, the price per dozen, or the cost per occupied bed as worked out under the present method. The true criterion is cost of laundry, plus cost of renewal of linen. But this is an instance of a department the cost of which should be viewed over a longer term than a year. Linen is not renewed annually in even approximately equal quantities. An average annual cost of renewals based on a period of five years, plus the year's laundry charges, and given that the standing charges and the division of in- and out-patient expenditure are calculated on identical lines, would give us statistics well worth the trouble of comparison.

If we prepare our figures on this suggested basis most of us would expect to find that the system of the hospital running its own laundry will in the end, given careful management, compare very favourably with any other method of administering the department, not only as regards cost, but in respect of comfort, punctuality of deliveries, and general efficiency. Here, too, is an opportunity for the consideration of hospital secretaries who are interested in co-operation, for we could point to a part of London where there are eight fair-sized hospitals within a square mile which could combine with every prospect of success in the management of a joint laundry.

